# A Micromorphic Beam Theory for Beams with Elongated Microstructures

**DOI:** 10.1038/s41598-020-64542-y

**Published:** 2020-05-14

**Authors:** M. Shaat, E. Ghavanloo, S. Emam

**Affiliations:** 1 Mechanical Engineering Department, Abu Dhabi University, Al Ain, P.O.BOX 1790, United Arab Emirates; 2 School of Mechanical Engineering, Shiraz University, Shiraz, 71963-16548 Iran; 3 Faculty of Engineering, Zagazig University, Zagazig, 44519 Egypt; 4 Department of Mechanical Engineering, American University of Sharjah, Sharjah, 26666 United Arab Emirates

**Keywords:** Mechanical engineering, Applied physics

## Abstract

A novel micromorphic beam theory that considers the exact shape and size of the beam’s microstructure is developed. The new theory complements the beam theories that are based on the classical mechanics by modeling the shape and size of the beam’s microstructure. This theory models the beam with a microstructure that has shape and size and exhibits microstrains that are independent of the beam’s macroscopic strains. This theory postulates six independent degrees of freedom to describe the axial and transverse displacements and the axial and shear microstrains of the beam. The detailed variational formulation of the beam theory is provided based on the reduced micromorphic model. For the first time, the displacement and microstrain fields of beams with elongated microstructures are developed. In addition, six material constants are defined to fully describe the beam’s microscopic and macroscopic stiffnesses, and two length scale parameters are used to capture the beam size effect. A case study of clamped-clamped beams is analytically solved to show the influence of the beam’s microstructural stiffness and size on its mechanical deformation. The developed micromorphic beam theory would find many important applications including the mechanics of advanced beams such as meta-, phononic, and photonic beams.

## Introduction

Various beam theories have been developed based on the classical theory of mechanics. In these theories, the beam is a colony of an infinite number of particles each of which is a mass point. The beam exhibits a displacement field that describes the motion of a particle in a two-dimensional domain. In Euler-Bernoulli beam theory, the displacement field is expressed in terms of the axial and transverse displacements of the beam’s mid-plane, and it assumes that the normal to the mid-plane remains undeformed and normal to the mid-plane after deformation^1–4^. The Euler-Bernoulli beam theory is limited to slender beams with high length-to-thickness ratio where the transverse shear effect is neglected. It was revealed that the Euler-Bernoulli beam theory is applicable for beams with length-to-thickness ratio of 20 and above depending on the material^1–4^. For thick beams, the Euler-Bernoulli beam theory underestimates the deflection (i.e., the transverse displacement of the mid-plane) and overestimates the natural frequencies^1–5^. Timoshenko beam theory outweighs the Euler-Bernoulli beam theory by accounting for the transverse shear strain and the rotary inertia effects^1,2,6^. The displacement field of the Timoshenko beam theory is expressed assuming that the normal to the mid-plane can rotate independently, but it remains straight after deformation^1,2,6^. More advanced beam theories that account for the distortion of the normal to the mid-plane have been developed^4,5,7^.

With the development of advanced materials, beams with independent microstructure are developed. The microstructure of these advanced beams would rotate and/or deform. These beams gave exceptional dynamical characteristics^8–12^. For instance, metamaterial beams with microstructural vibration absorbers were demonstrated with negative effective mass and stiffness^9,11^. In addition, the band gap behavior and vibration characteristics of metamaterial beams with microstructural resonances were studied^10,12^. Metamaterial beams with interconnected resonators were developed to give the flexural frequencies with two band gaps^8^. The exceptional characteristics of these beams have been demonstrated using lattice mechanics and lumped-models of the advanced beam dynamics^8–12^. Lumped-models can effectively reveal the topological properties of beams with microstructures. Nonetheless, other characteristics of these advanced beams would require modeling the beam as a distributed system, and, therefore, a continuum mechanics-based beam theory is required. These advanced beams exceeded the limits of applicability of the existing beam theories. Investigations of the mechanics of these beams based on the existing beam theories would lead to wrong estimations. In addition, the nontraditional characteristics, such as negative stiffness and band gaps^13–18^, cannot be captured by the classical beam theories.

Here, we develop a novel beam theory, based on the reduced micromorphic theory^18^, that represents the beam with an independent microstructure. The micromorphic theory instills the idea of independent microstructures^18–23^. In the context of a micromorphic model, the material is a composition of an infinite number of particles each of which can move, rotate, and deform^18,20,21^. The particle represents the building-block of the microstructure and exhibits 12 degrees of freedom: 3 displacements, 3 rotations, and 6 microstrains in the framework of the general micromorphic theory^20,21,23^. The constitutive equations of the general micromorphic theory depend on 18 material coefficients for an isotropic-linear elastic material^18,19,23^. Shaat^18^ has demonstrated the possibility of capturing all microstructural deformation patterns that would produce due to the 12 degrees of freedom of the particle by a reduced version of the micromorphic model. Therefore, the reduced micromorphic model^18^ was developed depending only on 8 material coefficients for an isotropic-linear elastic material. In the framework of the reduced micromorphic model, the particle exhibits three displacements (*u*_*i*_) and six independent microstrains (*s*_*ij*_). According to the reduced micromorphic model, these nine degrees of freedom are enough to represent all the microstructural deformation patterns^18^.

It should be mentioned that the nonlocal theories^24,25^, couple stress theories^26,27^, and strain-gradient theories^28,29^ have been extensively used to developed beam models that incorporate measures of the beam size effects. Beam models that are based on these theories adopted the displacement fields of the classical beam theories, and the parameters of the beam size effect were incorporated into the beam’s constitutive equations. Whereas these models have effectively related the beam mechanics to its size, the microstructure-dependence of the beam mechanics exceeds their limit of applicability. Therefore, more generalized theories such as micromorphic and micropolar theories are recommended to capture the beam mechanics in relation to its independent microstructure. These theories have shown an exceptional ability to capture the nontraditional phenomena of advanced materials, e.g., the band gap structure of phononic materials^18,30–32^, mechanics of multiscale materials^18,33^, and acoustic and optical properties of crystals^34,35^. However, their implementations to the mechanics of advanced structures, e.g., beams, are scarce. A few studies used the micromorphic theory for the electroelastic bending of piezoelectric beams^36^, the finite element modeling of micromorphic continua^37–39^, the static deformation of geometrically nonlinear micromorphic shells^40,41^, and the static bending of micropolar beams^42,43^.

In this study, we develop a novel micromorphic beam theory. This theory is developed based on the reduced micromorphic model^18^. Unlike the classical beam theories, the micromorphic beam theory accounts for the shape and size of the beam microstructure. In addition, the micromorphic beam theory outweighs existing beam models that depended on the nonlocal, couple stress, and strain gradient theories. The micromorphic beam theory accounts for not only the beam size effects but also the influence of the shape and size of the beam microstructure. For the first time, we formulate the degrees of freedom of beams with elongated microstructures, e.g., grains. These elongated grains exhibit six independent degrees of freedom. Thus, the micromorphic beam theory postulates six independent degrees of freedom that describe the axial and transverse displacements and the axial and shear microstrains of the beam. In addition, we provide an analytical solution of boundary value problems of micromorphic continua, for the first time. Analytical solutions for the elastostatic of micromorphic beams are provided.

The paper is organized as follows. First, the reduced micromorphic model is reviewed in Section 2. Then, the detailed variational formulation of the micromorphic beam theory is provided in the same section. To show the applicability of the micromorphic beam theory, the elastostatic behavior of clamped-clamped beams is modeled in Section 3. In Section 4, a parametric study on the influence of the microstructure topology dependence of the beam mechanics is provided.

## Micromorphic Beam Theory

In the context of the classical mechanics, the material is a composition of an infinite number of material particles, each of which is a mass point that can only move with no rotation or deformation. Whereas the particle represents the building-block of the material’s microstructure, the classical mechanics does not account for its shape or geometry. In contrast, in micromorphic mechanics, the material particle has a shape, and it does not only move but also rotates and/or deforms. The micromorphic mechanics instills the idea of microstructures with independent degrees of freedom. Given the aforementioned merits of the micromorphic mechanics over the classical mechanics, a novel micromorphic beam theory is developed here.

### Reduced micromorphic model: review

The original micromorphic theory, developed by Eringen^20,21^, represents a micromorphic continuum with 12 degrees of freedom. Thus, the equilibrium of the continuum under surface tractions is governed by 12 equations, and the constitutive equations depend on 18 material coefficients for homogeneous-isotropic linear elastic materials. By modeling the microstructure of a micromorphic material, Shaat^18^ revealed some redundant degrees of freedom in the context of the original micromorphic theory. It was demonstrated that all the microstructural deformation patterns can be captured by less number of degrees of freedom. The elimination of the redundant degrees of freedom from the original micromorphic theory yielded the reduced micromorphic model^18^. Only 8 material coefficients are required to fully describe the microstructural deformation of a micromorphic continuum according to the reduced micromorphic model.

The reduced micromorphic model defines the kinematics of materials using the following kinematical variables^18^:

1 $${\varepsilon }_{ij}={\varepsilon }_{ji}=\frac{1}{2}({u}_{i,j}+{u}_{j,i})$$

2 $${\chi }_{ijk}={\chi }_{ikj}={s}_{jk,i}\,\& \,{\chi }_{ijj}=0$$

3 $${\gamma }_{ij}={\gamma }_{ji}={\varepsilon }_{ij}-{s}_{ij}\,,{\rm{i.e}}.,\,i,j=x,y,z$$

where ${\varepsilon }_{ij}$ is the strain tensor, which is the symmetric dyadic of the displacement gradient tensor *u*_*i,j*_. Because the microstrain field is independent, *γ*_*ij*_ is a dyadic that accounts for the difference between microstrain (*s*_*ij*_) and macro-strain (${\varepsilon }_{ij}$) fields. ${\chi }_{ijk}$ is a triadic tensor that represents the gradient of the microstrain dyadic (*s*_*ij*_).

For a linear elastic-isotropic material, the strain energy density function is defined according to the reduced micromorphic model, as follows^18^:

4 $$\begin{array}{rcl}W & = & \frac{1}{2}{\lambda }_{m}{s}_{ii}{s}_{jj}+{\mu }_{m}{s}_{ij}{s}_{ij}+\frac{1}{2}\lambda {\gamma }_{ii}{\gamma }_{jj}+\mu {\gamma }_{ij}{\gamma }_{ij}+{\lambda }_{c}{\gamma }_{ii}{s}_{jj}+2{\mu }_{c}{\gamma }_{ij}{s}_{ij}\\  &  & +\,\frac{1}{2}{\lambda }_{m}{\ell }_{1}^{2}({\chi }_{iik}{\chi }_{jkj}+{\chi }_{ijk}{\chi }_{jik})+{\mu }_{m}{\ell }_{2}^{2}{\chi }_{ijk}{\chi }_{ijk}\end{array}$$

and the constitutive equations that involve six material coefficients (*λ*, *μ*, *λ*_*m*_, *μ*_*m*_, *λ*_*c*_, and *μ*_*c*_) and two length scale parameters (${\ell }_{1}$ and ${\ell }_{2}$) are expressed as follows^18^:

5 $${t}_{ij}={\lambda }_{m}{s}_{ll}{\delta }_{ij}+2{\mu }_{m}{s}_{ij}+{\lambda }_{c}{\gamma }_{ll}{\delta }_{ij}+2{\mu }_{c}{\gamma }_{ij}$$

6 $${\tau }_{ij}=\lambda {\gamma }_{ll}{\delta }_{ij}+2\mu {\gamma }_{ij}+{\lambda }_{c}{s}_{ll}{\delta }_{ij}+2{\mu }_{c}{s}_{ij}$$

7 $${m}_{ijk}=\frac{1}{2}{\lambda }_{m}{\ell }_{1}^{2}({\chi }_{mmk}{\delta }_{ij}+{\chi }_{mmj}{\delta }_{ik}+{\chi }_{jik}+{\chi }_{kij})+{\mu }_{m}{\ell }_{2}^{2}{\chi }_{ijk}$$

where *t*_*ij*_ and ${\tau }_{ij}$ are two symmetric stress dyadics, which measure the residual stresses in the elastic micromorphic material; *m*_*ijk*_ is a triadic that is conjugate to the microstrain gradient tensor (${\chi }_{ijk}$); *λ* and *μ* are the conventional Lame moduli; *λ*_*m*_ and *μ*_*m*_ are microstructural Lame moduli that describe the stiffness of the material’s microstructure; and *λ*_*c*_ and *μ*_*c*_ are coupling moduli used to capture the coupling between microstrains and macroscopic strains.

The dynamic equilibrium is governed by the following nine equations of motion^18^:

8 $${\tau }_{ji,j}+{f}_{i}=\rho {\ddot{u}}_{i}$$

9 $${m}_{ijk,i}+{\tau }_{jk}-{t}_{jk}+{H}_{jk}={\rho }_{m}J{\ddot{s}}_{jk}\,$$

with the following boundary conditions:

10 $${n}_{j}{\tau }_{ji}={ {\mathcal F} }_{i}$$

11 $${n}_{i}{m}_{ijk}={ {\mathcal H} }_{jk}\,$$

where $\rho$ is the material mass density; ${\rho }_{m}$ and *J* denote the particle’s mass density and micro-inertia, respectively; *f*_*i*_ and *H*_*jk*_ are body forces and body higher-order-moments, respectively; ${ {\mathcal F} }_{i}$ and ${ {\mathcal H} }_{jk}$ are surface forces and moments; and *n*_*i*_ is the unit normal.

The reduced micromorphic model is simple but is an effective approach to investigate the mechanics of micromorphic media. The model depends on eight material coefficients, which can be related to the material’s micro/macro-stiffnesses and microstructural topology. Recently, the material coefficients of the reduced micromorphic model were determined depending on the microstructure topology of different multiscale photonic materials and composite metamaterials^17,18^. In this study, the reduced micromorphic model is employed to develop a novel micromorphic beam theory.

### Microstructural degrees of freedom

**Figure 1:**
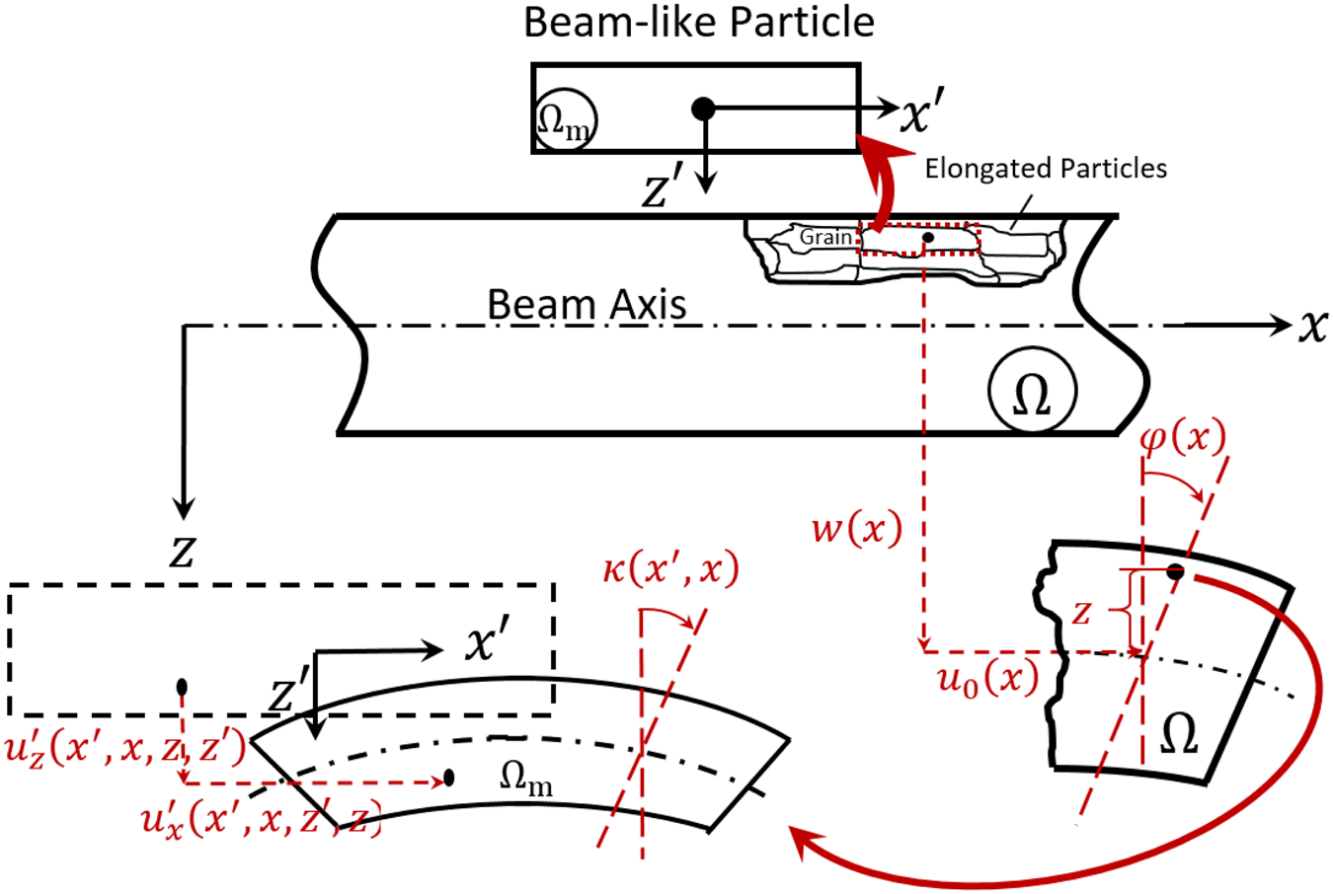
Deformation of micromorphic beams: Schematics of a beam with a deformed microstructure. The beam is a composition of elongated-particles that represent the beam granular microstructure. Two independent elastic domains are defined such that Ω(*x*, *y*, *z*) is the macroscopic domain, and Ω_m_(*x*′, *y*′, *z*′) is the microscopic domain of an elongated particle. Elongated particles are modeled as micro-beams exhibiting macroscopic displacements, ${u}_{x}(x,z)$ and ${u}_{z}(x)$, and micro-displacements, ${u}_{x}^{{\rm{{\prime} }}}(x{\rm{{\prime} }},x,z{\rm{{\prime} }},z)$ and ${u}_{z}^{{\rm{{\prime} }}}(x{\rm{{\prime} }},x,z{\rm{{\prime} }},z)$. The micro-beam (i.e., elongated-particle) deforms such that its cross section rotates with an angle, $\kappa (x{\rm{{\prime} }},x)$.

Here, the elastic beam is represented with a microstructure, as shown in Fig. 1. Two independent elastic domains are considered, $\Omega (x,y,z)$ and ${\Omega }_{{\rm{m}}}(x{\rm{{\prime} }},y{\rm{{\prime} }},z{\rm{{\prime} }})$, for the elastic beam and its microstructure, respectively. Beams are usually produced by rolling where grains are elongated in the rolling direction. Therefore, elongated-particles (beam-like particles) are considered to represent the beam microstructure (Fig. 1). An elongated-particle moves with *u*_*x*_ and *u*_*z*_ along *x* and *z*-directions, respectively (Fig. 1). The beam can be modeled such that the normal to its mid-plane remains straight after deformation, according to Timoshenko beam assumptions. Therefore, the displacements, *u*_*x*_ and *u*_*z*_, can be expressed as follows:

12 $${u}_{x}(x,z,t)={u}_{0}(x,t)-z\varphi (x,t)\,;{u}_{y}=0;\,{u}_{z}(x,t)=w(x,t)\,\forall (x,z)\in \Omega $$

where ${u}_{0}(x,t)$ and $w(x,t)$ are, respectively, the axial and transverse displacements of an elongated-particle located on the beam axis (i.e., $X=(x,0,0)$), and $\varphi (x,t)$ is the angle of rotation of the beam’s cross-section about *y-*axis with respect to *z*-axis.

In addition to the macroscopic displacements, *u*_*x*_ and *u*_*z*_, the elongated particle deforms exhibiting microstrains. These microstrains are produced due to a micro-displacement field, ${u}_{i}^{{\rm{{\prime} }}}(x{\rm{{\prime} }},z{\rm{{\prime} }},x,z,t)$, of a point belongs to the particle’s microscopic domain, Ω_m_. The micro-displacement, ${u}_{i}^{{\rm{{\prime} }}}$, is a slowly varying field over the microscopic domain, Ω_m_. When it is observed from the macroscopic domain Ω, the micro-displacement is a fast varying field. Therefore, it can be decomposed as follows:

13 $${u}_{i}^{{\rm{{\prime} }}}(x{\rm{{\prime} }},z{\rm{{\prime} }},x,z,t)={f}_{j}(x{\rm{{\prime} }},z{\rm{{\prime} }}){\psi }_{ji}(x,z,t)\,{\rm{\forall }}(x{\rm{{\prime} }},z{\rm{{\prime} }})\in {\Omega }_{{\rm{m}}}\,{\rm{\& }}\,{\rm{\forall }}(x,z)\in \Omega $$

where ${f}_{j}({x}^{{\rm{{\prime} }}},{z}^{{\rm{{\prime} }}})$ is a linear function that can be defined such that ${{\rm{\partial }}}^{2}{f}_{j}({x}^{{\rm{{\prime} }}},{z}^{{\rm{{\prime} }}})/{\rm{\partial }}{x}^{{\rm{{\prime} }}2}=0$ and ${{\rm{\partial }}}^{2}{f}_{j}({x}^{{\rm{{\prime} }}},{z}^{{\rm{{\prime} }}})/{\rm{\partial }}{z}^{{\rm{{\prime} }}2}=0$ ^19^. For linear elastic microstructures, it can be defined as ${f}_{j}({x}^{{\rm{{\prime} }}},{z}^{{\rm{{\prime} }}})={x}^{{\rm{{\prime} }}}+{z}^{{\rm{{\prime} }}}$. ${\psi }_{ji}$ is a fast-varying field within the macroscopic domain Ω, which is introduced as a micro-deformation tensor^19^.

As previously mentioned, the beam microstructure is a composition of elongated-particles, each of which can be modeled as a micro-beam. Therefore, the micro-deformation field can be defined as follows:

14 $$\begin{array}{ccc}{\psi }_{xx}(x,z,t) & = & \frac{{\rm{\partial }}{u}_{x}^{{\rm{{\prime} }}}}{{\rm{\partial }}x{\rm{{\prime} }}}={s}_{0}(x,t)-z{\theta }_{,x}(x,t)\,;\,{\psi }_{zx}(x,t)=\frac{{\rm{\partial }}{u}_{x}^{{\rm{{\prime} }}}}{{\rm{\partial }}z{\rm{{\prime} }}}\\  & = & \theta (x,t)\,;\,{\psi }_{xz}(x,t)=\frac{{\rm{\partial }}{u}_{z}^{{\rm{{\prime} }}}}{{\rm{\partial }}x{\rm{{\prime} }}}={p}_{,x}(x,t)\end{array}$$

where ${s}_{0}(x,t)$, $p(x,t)$, and $\theta (x,t)$ are fast-varying functions that map microscopic fields, e.g., $\kappa (x{\rm{{\prime} }},x)$ which is the micro-rotation of the cross-section of the micro-beam, to the macroscopic domain, Ω.

According to the reduced micromorphic model, all microstructural deformations can be detected utilizing a micro-strain tensor, which is the symmetric part of the micro-deformation tensor *ψ*_*ij*_ ^18^. According to Eq. (14), the non-zero components of the microstrain tensor can be derived as follows:

15 $$\begin{array}{llll}{s}_{xx}(x,z,t) & = & {\psi }_{xx}={s}_{0}(x,t)-z{\theta }_{,x}(x,t) & \forall (x,z)\in \Omega \\ {s}_{xz}(x,t) & = & \frac{1}{2}({\psi }_{xz}+{\psi }_{zx})=\frac{1}{2}({p}_{,x}(x,t)-\theta (x,t)) & \forall x\in \Omega \end{array}$$

It follows from the preceding discussion that a micromorphic beam exhibits six independent degrees of freedom (i.e., *u*_0_, *w*, $\varphi$, *s*_0_, *p*, $\theta$). These degrees are generated due to four independent degrees of freedom of the beam microstructure (i.e., ${u}_{x}$, ${u}_{z}$, ${s}_{xx}$, ${s}_{xz}$). Thus, a particle located at $(x,y,z)$ exhibits displacements and microstrains defined by ${u}_{x}$, ${u}_{z}$, ${s}_{xx}$, and ${s}_{xz}$ in Eqs. (12) and (15).

### Kinematical variables

The kinematical variables that represent the beam deformation are formed based on the reduced micromorphic model. The substitution of Eqs. (12) and (14) into Eqs. (1–3) gives the non-zero components of the kinematical variables, ${\varepsilon }_{ij}$, ${\gamma }_{ij}$, and ${\chi }_{ijk}$, of the considered micromorphic beam with the form:

16 $$\begin{array}{rcl}{\varepsilon }_{xx}(x,z,t) & = & {u}_{0,x}(x,t)-z{\varphi }_{,x}(x,t)\\ {\varepsilon }_{xz}(x,t) & = & \frac{1}{2}({w}_{,x}(x,t)-\varphi (x,t))\end{array}$$

17 $$\begin{array}{rcl}{\gamma }_{xx}(x,z,t) & = & ({u}_{0,x}(x,t)-{s}_{0}(x,t))-z({\varphi }_{,x}(x,t)-{\theta }_{,x}(x,t))\\ {\gamma }_{xz}(x,t) & = & \frac{1}{2}({w}_{,x}(x,t)-{p}_{,x}(x,t))-\frac{1}{2}(\varphi (x,t)-\theta (x,t))\end{array}$$

18 $${\chi }_{xxz}(x,t)=\frac{1}{2}({p}_{,xx}(x,t)-{\theta }_{,x}(x,t))$$

### Constitutive equations

The constitutive equations of the developed micromorphic beam model are determined by substituting Eqs. (16–18) into Eqs. (5–7). Thus, the non-zero components of the different stress fields are obtained as follows:

19 $$\begin{array}{rcl}{t}_{xx}(x,z,t) & = & ({\lambda }_{m}+2{\mu }_{m}-{\lambda }_{c}-2{\mu }_{c}){s}_{0}-z({\lambda }_{m}+2{\mu }_{m}-{\lambda }_{c}-2{\mu }_{c}){\theta }_{,x}\\  &  & +\,({\lambda }_{c}+2{\mu }_{c}){u}_{0,x}-z({\lambda }_{c}+2{\mu }_{c}){\varphi }_{,x}\\ {t}_{xz}(x,t) & = & ({\mu }_{m}-{\mu }_{c})({p}_{,x}-\theta )+{\mu }_{c}({w}_{,x}-\varphi )\\ {t}_{zz}(x,z,t) & = & {t}_{yy}(x,z,t)=({\lambda }_{m}-{\lambda }_{c}){s}_{0}-z({\lambda }_{m}-{\lambda }_{c}){\theta }_{,x}+{\lambda }_{c}{u}_{0,x}-z{\lambda }_{c}{\varphi }_{,x}\end{array}$$

20 $$\begin{array}{rcl}{\tau }_{xx}(x,z,t) & = & ({\lambda }_{c}+2{\mu }_{c}-\lambda -2\mu ){s}_{0}-z({\lambda }_{c}+2{\mu }_{c}-\lambda -2\mu ){\theta }_{,x}\\  &  & +\,(\lambda +2\mu ){u}_{0,x}-z(\lambda +2\mu ){\varphi }_{,x}\\ {\tau }_{xz}(x,t) & = & ({\mu }_{c}-\mu )({p}_{,x}-\theta )+\mu ({w}_{,x}-\varphi )\\ {\tau }_{zz}(x,z,t) & = & {\tau }_{yy}(x,z,t)=({\lambda }_{c}-\lambda ){s}_{0}-z({\lambda }_{c}-\lambda ){\theta }_{,x}+\lambda {u}_{0,x}-z\lambda {\varphi }_{,x}\end{array}$$

21 $$\begin{array}{rcl}{m}_{zxx} & = & \frac{1}{2}{\lambda }_{m}{\ell }_{1}^{2}({p}_{,xx}-{\theta }_{,x})\\ {m}_{xxz} & = & {m}_{xzx}=\frac{1}{2}\left({\lambda }_{m}{\ell }_{1}^{2}+\frac{1}{2}{\mu }_{m}{\ell }_{2}^{2}\right)({p}_{,xx}-{\theta }_{,x})\\ {m}_{yyz} & = & {m}_{yzy}={m}_{zzz}=\frac{1}{4}{\lambda }_{m}{\ell }_{1}^{2}({p}_{,xx}-{\theta }_{,x})\end{array}$$

where ${\tau }_{ij}$ are the typical Cauchy-type stresses; *t*_*ij*_ are the components of the microstresses of the elongated microstructure of the beam; and *m*_*ijk*_ are the components of the double-stresses.

### Micromorphic beam material parameters

It follows from Eqs. (19–21) that the constitutive equations of the developed micromorphic beam theory depend only on 6 material coefficients and two length scales. These material coefficients can be defined for a specific beam with an elongated microstructure. *λ* and *μ* are the classical Lame moduli of the beam. These moduli can be determined based on the typical flexural bending of beams test. *λ*_*m*_ and *μ*_*m*_ are the Lame moduli of the beam’s microstructure. A typical example of a beam with an elongated microstructure is a polycrystalline material beam made by rolling. The stiffness of the grains of a polycrystalline material is usually considered the same as the stiffness of the entire material. In this case, the Lame moduli, *λ*_*m*_ and *μ*_*m*_, would be considered the same as those of the classical Lame moduli of the beam, i.e., ${\lambda }_{m}=\lambda$ and ${\mu }_{m}=\mu$. However, for the case that the beam microstructure is of a different stiffness, the Lame moduli of the microstructure would be independently defined. The Lame moduli of the microstructure can be determined experimentally by the direct testing a single crystal of the beam material, or by testing the shifts in the natural frequencies of the beam, as we decrease its size^44^.

In addition to the aforementioned Lame moduli, *λ*_*c*_ and *μ*_*c*_ are introduced to account for the difference in the deformation and stress fields between the elongated microstructure and the entire beam. These special Lame moduli depend on the stiffness of the boundary of the elongated microstructure, e.g., stiffness of the grain boundary. Indeed, the microstress field of the grains depends on the grain boundary stiffness and how it would slide to allow for the grain deformation. According to the preceding equations, the transmissibility of the external stresses through the grain boundary to the grain can be measured by $(1-{\lambda }_{c}/\lambda )\times 100$ and $(1-{\mu }_{c}/\mu )\times 100$. When ${\lambda }_{c}=0$ and ${\mu }_{c}=0$, the transmissibility of the grain boundary is 100%, which indicates no difference between the microdeformation of the grain and the deformation of the entire beam.

The developed beam model account not only for the effects of the beam microstructure but also the beam size effects. The size effects of the beam are captured via the two length scales, ${\ell }_{1}$ and ${\ell }_{2}$. These length parameters mainly scale the dependence of the beam mechanics on its microstructural deformations. Thus, the length parameters can be considered to scale the microstructural stiffness to the macro-stiffness of the beam, i.e., ${\ell }_{1}=\sqrt{\lambda /{\lambda }_{m}}$ and ${\ell }_{2}=\sqrt{\mu /{\mu }_{m}}$. On the other hand, these length parameters would be defined depending on the microstructure size-to-the beam size ratio. Thus, for a macroscopic beam with infinitesimal microstructural grains, ${\ell }_{1}\to 0$ and ${\ell }_{2}\to 0$. These length scales can be experimentally determined by measuring the shifts in the frequencies or the transmission phase of the beam, as we decrease its size^44,45^.

### Equations of motion

In light of the defined kinematical variables (Eqs. (16–18)) and the derived constitutive Eqs. (19–21) of the micromorphic beam, the first variation of the deformation energy of the beam can be written in the form:

22 $$\delta U={\int }_{x=0}^{L}{\int }_{A}({\tau }_{xx}\delta {\gamma }_{xx}+2{\tau }_{xz}\delta {\gamma }_{xz}+{t}_{xx}\delta {s}_{xx}+2{t}_{xz}\delta {s}_{xz}+2{m}_{xxz}\delta {\chi }_{xxz})dAdx$$

where *L* is the beam length, and *A* is the beam cross-sectional area. The substitution of Eqs. (16–21) into Eq. (22) yields:

23 $$\begin{array}{rcl}\delta U & = & {\int }_{x=0}^{L}[\,-\,{N}_{xx,x}\delta {u}_{0}+(-{N}_{xx}+{Y}_{xx})\delta {s}_{0}-{N}_{xz,x}\delta w+({M}_{xx,x}-{N}_{xz})\delta \varphi \\  &  & +({N}_{xz,x}-{Y}_{xz,x}+{\eta }_{xxz,xx})\delta p+(-{M}_{xx,x}+{R}_{xx,x}+{N}_{xz}-{Y}_{xz}+{\eta }_{xxz,x})\delta \theta ]dx\\  &  & +{{N}_{xx}\delta {u}_{0}|}_{0}^{L}+{{N}_{xz}\delta w|}_{0}^{L}-{{M}_{xx}\delta \varphi |}_{0}^{L}+{({Y}_{xz}-{N}_{xz}-{\eta }_{xxz,x})\delta p|}_{0}^{L}\\  &  & +{{\eta }_{xxz}\delta ({p}_{,x})|}_{0}^{L}+{({M}_{xx}-{R}_{xx}-{\eta }_{xxz})\delta \theta |}_{0}^{L}\end{array}$$

where the resultants introduced in Eq. (23) are defined as follows:

24 $$\begin{array}{rcl}{N}_{xx} & = & {\int }_{A}{\tau }_{xx}dA;{N}_{xz}={\int }_{A}{\tau }_{xz}dA;{M}_{xx}={\int }_{A}z{\tau }_{xx}dA\\ {Y}_{xx} & = & {\int }_{A}{t}_{xx}dA;{Y}_{xz}={\int }_{A}{t}_{xz}dA;{R}_{xx}={\int }_{A}z{t}_{xx}dA\\ {\eta }_{xxz} & = & {\int }_{A}{m}_{xxz}dA\end{array}$$

The explicit form of these resultants in terms of the displacement and microstrain fields are expressible in the form:

25 $$\begin{array}{rcl}{N}_{xx} & = & {\alpha }_{0}{u}_{0,x}+{\alpha }_{1}{s}_{0}\\ {N}_{xz} & = & {\beta }_{0}{w}_{,x}-{\beta }_{0}\varphi +{\beta }_{1}{p}_{,x}-{\beta }_{1}\theta \\ {M}_{xx} & = & -{\bar{\alpha }}_{0}{\varphi }_{,x}-{\bar{\alpha }}_{1}{\theta }_{,x}\\ {Y}_{xx} & = & ({\alpha }_{1}+{\alpha }_{0}){u}_{0,x}-{\alpha }_{2}{s}_{0}\\ {Y}_{xz} & = & -({\beta }_{1}+{\beta }_{0})\varphi +{\beta }_{2}\theta +({\beta }_{1}+{\beta }_{0}){w}_{,x}-{\beta }_{2}{p}_{,x}\\ {R}_{xx} & = & {\bar{\alpha }}_{2}{\theta }_{,x}-({\bar{\alpha }}_{1}+{\bar{\alpha }}_{0}){\varphi }_{,x}\\ {\eta }_{xxz} & = & {\gamma }_{1}({p}_{,xx}-{\theta }_{,x})\end{array}$$

where

26 $$\begin{array}{rcl}{\alpha }_{0} & = & (\lambda +2\mu )A;{\alpha }_{1}=({\lambda }_{c}+2{\mu }_{c}-\lambda -2\mu )A;{\alpha }_{2}=({\lambda }_{c}+2{\mu }_{c}-{\lambda }_{m}-2{\mu }_{m})A\\ {\bar{\alpha }}_{0} & = & (\lambda +2\mu )I;{\bar{\alpha }}_{2}=({\lambda }_{c}+2{\mu }_{c}-{\lambda }_{m}-2{\mu }_{m})I;{\bar{\alpha }}_{1}=({\lambda }_{c}+2{\mu }_{c}-\lambda -2\mu )I\\ {\beta }_{0} & = & \mu A;{\beta }_{1}=({\mu }_{c}-\mu )A;{\beta }_{2}=({\mu }_{c}-{\mu }_{m})A\\ \gamma  & = & \frac{1}{2}({\lambda }_{m}{\ell }_{1}^{2}+{\mu }_{m}{\ell }_{2}^{2})A\end{array}$$

where *I* is the beam’s area moment of inertia, i.e., $I={\int }_{A}{z}^{2}dA$.

Similar to the deformation energy, the first variation of the kinetic energy, *δK*, is determined as follows:

27 $$\begin{array}{rcl}\delta K & = & -{\int }_{x=0}^{L}(\rho A{\ddot{u}}_{0}\delta {u}_{0}+\rho I\ddot{\varphi }\delta \varphi +\rho A\ddot{w}\delta w+{\rho }_{m}JA{\ddot{s}}_{0}\delta {s}_{0}-{\rho }_{m}JI{\ddot{\theta }}_{,xx}\delta \theta \\  &  & -\frac{1}{4}{\rho }_{m}JA{\ddot{p}}_{,xx}\delta p+\frac{1}{4}{\rho }_{m}JA{\ddot{\theta }}_{,x}\delta p-\frac{1}{4}{\rho }_{m}JA{\ddot{p}}_{,x}\delta \theta +\frac{1}{4}{\rho }_{m}JA\ddot{\theta }\delta \theta )dV\end{array}$$

In addition, the first variation of the work done by body forces and surface tractions can be defined, as follows:

28 $$\begin{array}{rcl}\delta Q & = & {\int }_{V}({f}_{x}(x,z,t)\delta {u}_{x}(x,z,t)+{f}_{z}(x,t)\delta {u}_{z}(x,t)+{H}_{xx}(x,z,t)\delta {s}_{xx}\\  &  & +\,{H}_{xz}(x,t)\delta {s}_{xz}(x,t))dV+{\int }_{S}({ {\mathcal F} }_{x}\delta {u}_{x}+{ {\mathcal F} }_{z}\delta {u}_{z}+{ {\mathcal H} }_{xx}\,\delta {s}_{xx}+{ {\mathcal H} }_{xz}\,\delta {s}_{xz})dS\end{array}$$

The substitution of Eqs. (12) and (15) into Eq. (28) gives the work done in the form:

29 $$\begin{array}{rcl}\delta Q & = & {\int }_{x=0}^{L}({q}_{x}(x,t)\delta {u}_{0}(x,t)-{m}_{x}(x,t)\delta \varphi (x,t)+{q}_{z}(x,t)\delta w(x,t)\\  &  & +\,{h}_{xx}(x,t)\delta {s}_{0}(x,t)-{v}_{xx}(x,t)\delta {\theta }_{,x}(x,t)+\frac{1}{2}{h}_{xz}(x,t)\delta {p}_{,x}(x,t)\\  &  & -\frac{1}{2}{h}_{xz}(x,t)\delta \theta (x,t))dx+{{{\rm{Q}}}_{x}\delta {u}_{0}|}_{0}^{L}-{{ {\mathcal M} }_{x}\delta \varphi |}_{0}^{L}+{{{\rm{Q}}}_{z}\delta w|}_{0}^{L}\\  &  & +{{{\mathscr{g}}}_{xx}\,\delta {s}_{0}|}_{0}^{L}-{{ {\mathcal R} }_{xx}\,\delta {\theta }_{,x}|}_{0}^{L}+{\frac{1}{2}{{\mathscr{g}}}_{xz}\delta {p}_{,x}|}_{0}^{L}-{\frac{1}{2}{{\mathscr{g}}}_{xz}\delta \theta |}_{0}^{L}\end{array}$$

where

30 $$\begin{array}{rcl}{q}_{x} & = & {\int }_{A}{f}_{x}dA;{m}_{x}={\int }_{A}z{f}_{x}dA;{q}_{z}={\int }_{A}{f}_{z}dA\\ {h}_{xx} & = & {\int }_{A}{H}_{xx}dA;{v}_{xx}={\int }_{A}z{H}_{xx}dA;{h}_{xz}={\int }_{A}{H}_{xz}dA\\ {{\rm{Q}}}_{x} & = & {\int }_{A}{ {\mathcal F} }_{x}dA;{{\rm{Q}}}_{z}={\int }_{A}{ {\mathcal F} }_{z}dA;{ {\mathcal M} }_{x}={\int }_{A}z{ {\mathcal F} }_{x}dA;{{\mathscr{g}}}_{xx}\\  &  & ={\int }_{A}{ {\mathcal H} }_{xx}dA;{ {\mathcal R} }_{xx}={\int }_{A}z{ {\mathcal H} }_{xx}dA;{{\mathscr{g}}}_{xz}={\int }_{A}{ {\mathcal H} }_{xz}dA\end{array}$$

The equations of motion are obtained by employing Hamilton’s principle, ${\int }_{0}^{t}(\delta Q+\delta K-\delta U)dt=0$, and then setting the coefficients of *δu*_0_, *δw*, $\delta \varphi$, $\delta {s}_{0}$, $\delta p$, and $\delta \theta$ to zero:

31 $$\begin{array}{c}\delta {u}_{0}:{N}_{xx,x}+{q}_{x}=\rho A{\ddot{u}}_{0}\\ \delta {s}_{0}:\,{N}_{xx}-{Y}_{xx}+{h}_{xx}={\rho }_{m}JA{\ddot{s}}_{0}\\ \delta w:\,{N}_{xz,x}+{q}_{z}=\rho A\ddot{w}\\ \delta \varphi :\,-{M}_{xx,x}+{N}_{xz}-{m}_{x}=\rho I\ddot{\varphi }\\ \delta p:\,-{N}_{xz,x}+{Y}_{xz,x}-{\eta }_{xxz,xx}-\frac{1}{2}{h}_{xz,x}=-\frac{1}{4}{\rho }_{m}JA{\ddot{p}}_{,xx}+\frac{1}{4}{\rho }_{m}JA{\ddot{\theta }}_{,x}\\ \delta \theta :\,{M}_{xx,x}-{R}_{xx,x}-{N}_{xz}+{Y}_{xz}-{\eta }_{xxz,x}+{v}_{xx,x}-\frac{1}{2}{h}_{xz}=-{\rho }_{m}JI{\ddot{\theta }}_{,xx}-\frac{1}{4}{\rho }_{m}JA{\ddot{p}}_{,x}+\frac{1}{4}{\rho }_{m}JA\ddot{\theta }\end{array}$$

with the boundary conditions:

32 $$\begin{array}{c}-{N}_{xx}+{Q}_{x}=0\,{\rm{or}}\,{u}_{0}={U}_{0}\\ -{N}_{xz}+{{\rm{Q}}}_{z}=0\,{\rm{or}}\,w=W\\ {M}_{xx}-{ {\mathcal M} }_{x}=0\,{\rm{or}}\,\varphi =\Phi \\ -{Y}_{xz}+{N}_{xz}+{\eta }_{xxz,x}-\frac{1}{2}{{\mathscr{g}}}_{xz,x}=0\,{\rm{or}}\,p=P\\ -{\eta }_{xxz}+\frac{1}{2}{{\mathscr{g}}}_{xz}=0\,{\rm{or}}\,{p}_{,x}={P}_{,x}\\ -{M}_{xx}+{R}_{xx}+{\eta }_{xxz}+{ {\mathcal R} }_{xx,x}-\frac{1}{2}{{\mathscr{g}}}_{xz}=0\,{\rm{or}}\,\theta =\Theta \end{array}$$

where ${u}_{0}$, $W$, $\Phi$, $P$, *P*_,__*x*_, and $\Theta$ are prescribed values of the different degrees of freedom of the beam.

Equations of motion in terms of the displacement and microstrain fields are obtained by substituting Eq. (25) into Eq. (31), as follows:

33 $${\alpha }_{0}{u}_{0,xx}+{\alpha }_{1}{s}_{0,x}+{q}_{x}=\rho A{\ddot{u}}_{0}$$

34 $${\bar{\alpha }}_{0}{\varphi }_{,xx}+{\bar{\alpha }}_{1}{\theta }_{,xx}+{\beta }_{0}{w}_{,x}+{\beta }_{1}{p}_{,x}-{\beta }_{0}\varphi -{\beta }_{1}\theta -{m}_{x}=\rho I\ddot{\varphi }$$

35 $${\beta }_{0}{w}_{,xx}+{\beta }_{1}{p}_{,xx}-{\beta }_{0}{\varphi }_{,x}-{\beta }_{1}{\theta }_{,x}+{q}_{z}=\rho A\ddot{w}$$

36 $$-{\alpha }_{1}{u}_{0,x}+({\alpha }_{1}+{\alpha }_{2}){s}_{0}+{h}_{xx}={\rho }_{m}JA{\ddot{s}}_{0}$$

37 $$\begin{array}{c}-\gamma {p}_{,xxx}+\gamma {\theta }_{,xx}+{\bar{\alpha }}_{1}{\varphi }_{,xx}-({\bar{\alpha }}_{1}+{\bar{\alpha }}_{2}){\theta }_{,xx}+{\beta }_{1}{w}_{,x}-({\beta }_{1}+{\beta }_{2}){p}_{,x}-{\beta }_{1}\varphi \\ +\,({\beta }_{1}+{\beta }_{2})\theta +{v}_{xx,x}-\frac{1}{2}{h}_{xz}=-{\rho }_{m}JI{\ddot{\theta }}_{,xx}-\frac{1}{4}{\rho }_{m}JA{\ddot{p}}_{,x}+\frac{1}{4}{\rho }_{m}JA\ddot{\theta }\end{array}$$

38 $$\begin{array}{c}-\gamma {p}_{,xxxx}+\gamma {\theta }_{,xxx}+{\beta }_{1}{w}_{,xx}-({\beta }_{1}+{\beta }_{2}){p}_{,xx}-{\beta }_{1}{\varphi }_{,x}+({\beta }_{1}+{\beta }_{2}){\theta }_{,x}\\ -\frac{1}{2}{h}_{xz,x}=-\frac{1}{4}{\rho }_{m}JA{\ddot{p}}_{,xx}+\frac{1}{4}{\rho }_{m}JA{\ddot{\theta }}_{,x}\end{array}$$

Similarly, the boundary conditions (Eq. (32)) can be written in terms of the displacement and microstrain fields, as follows:

39 $$\begin{array}{c}{\alpha }_{0}{u}_{0,x}+{\alpha }_{1}{s}_{0}={Q}_{x}\,{\rm{or}}\,{u}_{0}={U}_{0}\\ {\beta }_{0}{w}_{,x}-{\beta }_{0}\varphi +{\beta }_{1}{p}_{,x}-{\beta }_{1}\theta ={{\rm{Q}}}_{z}\,{\rm{or}}\,w=W\\ -{\bar{\alpha }}_{0}{\varphi }_{,x}-{\bar{\alpha }}_{1}{\theta }_{,x}={ {\mathcal M} }_{x}\,{\rm{or}}\,\varphi =\Phi \\ ({\beta }_{1}+{\beta }_{0})\varphi +{\beta }_{2}\theta -({\beta }_{1}+{\beta }_{0}){w}_{,x}+{\beta }_{2}{p}_{,x}+{\beta }_{0}{w}_{,x}-{\beta }_{0}\varphi +{\beta }_{1}{p}_{,x}-{\beta }_{1}\theta \\ +\gamma ({p}_{,xxx}-{\theta }_{,xx})=\frac{1}{2}{{\mathscr{g}}}_{xz,x}\,{\rm{or}}\,p=P\\ \gamma ({p}_{,xx}-{\theta }_{,x})=\frac{1}{2}{{\mathscr{g}}}_{xz}\,{\rm{or}}\,{p}_{,x}={P}_{,x}\\ {\bar{\alpha }}_{0}{\varphi }_{,x}+{\bar{\alpha }}_{1}{\theta }_{,x}+{\bar{\alpha }}_{2}{\theta }_{,x}-({\bar{\alpha }}_{1}+{\bar{\alpha }}_{0}){\varphi }_{,x}+\gamma ({p}_{,xx}-{\theta }_{,x})+{\bar{\alpha }}_{2}{\theta }_{,xx}-({\bar{\alpha }}_{1}+{\bar{\alpha }}_{0}){\varphi }_{,xx}\\ =\,\frac{1}{2}{{\mathscr{g}}}_{xz}\,{\rm{or}}\,\theta =\Theta \end{array}$$

### Elastostatics of micromorphic beams

In this section, the derived micromorphic theory is applied to model the elastostatic behavior of micromorphic beams. The equations of the elastostatic equilibrium and the boundary conditions are first derived. Then, the general solutions of the derived equations are presented. To show the applicability of the derived micromorphic beam theory, explicit solutions of clamped-clamped beams are determined.

### Elastostatic equilibrium

The governing equations of the elastostatic behavior of micromorphic beams under only axial and transverse distributed loads, ${q}_{x}(x)$ and ${q}_{z}(x)$, can be written by dropping the time-dependent terms in Eqs. (33–38), as follows:

40 $${\alpha }_{0}{u}_{0,xx}+{\alpha }_{1}{s}_{0,x}+{q}_{x}=0$$

41 $${\bar{\alpha }}_{0}{\varphi }_{,xx}+{\bar{\alpha }}_{1}{\theta }_{,xx}+{\beta }_{0}{w}_{,x}+{\beta }_{1}{p}_{,x}-{\beta }_{0}\varphi -{\beta }_{1}\theta =0$$

42 $${\beta }_{0}{w}_{,xx}+{\beta }_{1}{p}_{,xx}-{\beta }_{0}{\varphi }_{,x}-{\beta }_{1}{\theta }_{,x}+{q}_{z}=0$$

43 $$-{\alpha }_{1}{u}_{0,x}+({\alpha }_{1}+{\alpha }_{2}){s}_{0}=0$$

44 $$-\gamma {p}_{,xxx}+\gamma {\theta }_{,xx}+{\bar{\alpha }}_{1}{\varphi }_{,xx}-({\bar{\alpha }}_{1}+{\bar{\alpha }}_{2}){\theta }_{,xx}+{\beta }_{1}{w}_{,x}-({\beta }_{1}+{\beta }_{2}){p}_{,x}-{\beta }_{1}\varphi +({\beta }_{1}+{\beta }_{2})\theta =0$$

45 $$-\gamma {p}_{,xxxx}+\gamma {\theta }_{,xxx}+{\beta }_{1}{w}_{,xx}-({\beta }_{1}+{\beta }_{2}){p}_{,xx}-{\beta }_{1}{\varphi }_{,x}+({\beta }_{1}+{\beta }_{2}){\theta }_{,x}=0$$

It follows from Eqs. (40–45) that the axial displacement and microstrain of the mid-plane, ${u}_{0}$ and ${s}_{0}$, are independent of the transverse ones. Therefore, explicit solutions for ${u}_{0}$ and ${s}_{0}$ can be obtained by simultaneously solving Eqs. (40) and (43). In this way, eliminating ${s}_{0}$ between Eqs. (40) and (43), we obtain:

46 $$\frac{{\alpha }_{1}^{2}+{\alpha }_{0}({\alpha }_{1}+{\alpha }_{2})}{({\alpha }_{1}+{\alpha }_{2})}{u}_{0,xx}+{q}_{x}(x)=0$$

The general solution of Eq. (46) is obtained as:

47 $${u}_{0}(x)={C}_{1}+{C}_{2}x-\left(\frac{{\alpha }_{1}+{\alpha }_{2}}{{\alpha }_{1}^{2}+{\alpha }_{0}({\alpha }_{1}+{\alpha }_{2})}\right){\int }_{0}^{x}(x-\xi ){q}_{x}(\xi )d\xi $$

where *C*_1_ and *C*_2_ are the integration constants. Substitution of Eq. (47) into Eq. (43), yields

48 $${s}_{0}(x)=\left(\frac{{\alpha }_{1}}{{\alpha }_{1}+{\alpha }_{2}}\right){C}_{2}-\left(\frac{{\alpha }_{1}}{{\alpha }_{1}^{2}+{\alpha }_{0}({\alpha }_{1}+{\alpha }_{2})}\right){\int }_{0}^{x}{q}_{x}(\xi )d\xi $$

Now, Eqs. (42) and (45) are integrated with respect to *x* to give:

49 $${\beta }_{0}{w}_{,x}+{\beta }_{1}{p}_{,x}-{\beta }_{0}\varphi -{\beta }_{1}\theta +{\int }_{0}^{x}{q}_{z}(\xi )d\xi ={C}_{3}$$

50 $$-\gamma {p}_{,xxx}+\gamma {\theta }_{,xx}+{\beta }_{1}{w}_{,x}-({\beta }_{1}+{\beta }_{2}){p}_{,x}-{\beta }_{1}\varphi +({\beta }_{1}+{\beta }_{2})\theta ={C}_{4}$$

in which *C*_3_ and *C*_4_ are two unknown constants. Substituting Eqs. (49) and (50) into Eqs. (41) and (44), the following equations are derived:

51 $${\bar{\alpha }}_{0}{\varphi }_{,xx}+{\bar{\alpha }}_{1}{\theta }_{,xx}+{C}_{3}={\int }_{0}^{x}{q}_{z}(\xi )d\xi $$

52 $${\bar{\alpha }}_{1}{\varphi }_{,xx}-({\bar{\alpha }}_{1}+{\bar{\alpha }}_{2}){\theta }_{,xx}+{C}_{4}=0$$

By eliminating ${\theta }_{,xx}$ between Eqs. (51) and (52), a differential equation in term of the variable $\varphi$ is derived as follows:

53 $${\varphi }_{,xx}=\frac{{\bar{\alpha }}_{1}({\bar{\alpha }}_{1}+{\bar{\alpha }}_{2})}{{\bar{\alpha }}_{1}^{2}+{\bar{\alpha }}_{0}({\bar{\alpha }}_{1}+{\bar{\alpha }}_{2})}\left(\frac{1}{{\bar{\alpha }}_{1}}{\int }_{0}^{x}{q}_{z}(\xi )d\xi -\left(\frac{{C}_{3}}{{\bar{\alpha }}_{1}}+\frac{{C}_{4}}{{\bar{\alpha }}_{1}+{\bar{\alpha }}_{2}}\right)\right)$$

The general solution of the differential Eq. (53) can be obtained as:

54 $$\varphi (x)=\frac{{\bar{\alpha }}_{1}({\bar{\alpha }}_{1}+{\bar{\alpha }}_{2})}{{\bar{\alpha }}_{1}^{2}+{\bar{\alpha }}_{0}({\bar{\alpha }}_{1}+{\bar{\alpha }}_{2})}\left(\frac{1}{{\bar{\alpha }}_{1}}{\int }_{0}^{x}{\int }_{0}^{\eta }(x-\xi ){q}_{z}(\xi )d\xi d\eta -\left(\frac{{C}_{3}}{{\bar{\alpha }}_{1}}+\frac{{C}_{4}}{{\bar{\alpha }}_{1}+{\bar{\alpha }}_{2}}\right)\frac{{x}^{2}}{2}\right)+{C}_{5}x+{C}_{6}$$

By integrating Eq. (52) twice, we obtain:

55 $$\theta (x)=\frac{1}{({\bar{\alpha }}_{1}+{\bar{\alpha }}_{2})}\left({\bar{\alpha }}_{1}\varphi +\frac{{C}_{4}{x}^{2}}{2}\right)+{C}_{7}x+{C}_{8}$$

Substituting *w*_,*x*_ from Eq. (49) into Eq. (50), integrating the resultant equation, and after making some simplifications, we get:

56 $${p}_{,xx}+{\kappa }^{2}p=H(x)$$

where

57 $${\kappa }^{2}=\frac{{\beta }_{1}^{2}+({\beta }_{1}+{\beta }_{2}){\beta }_{0}}{\gamma {\beta }_{0}}$$

58 $$H(x)=\left({\theta }_{,x}+\frac{({\beta }_{1}^{2}+{\beta }_{0}({\beta }_{1}+{\beta }_{2}))}{\gamma {\beta }_{0}}{\int }_{0}^{x}\theta (\xi )d\xi -\frac{({\beta }_{0}{C}_{4}-{C}_{3}{\beta }_{1})x}{\gamma {\beta }_{0}}-\frac{{\beta }_{1}}{\gamma {\beta }_{0}}{\int }_{0}^{x}(x-\xi ){q}_{z}(\xi )d\xi \right)+{C}_{9}$$

The general solution of Eq. (56) is given by:

59 $$p(x)={C}_{10}\,\sin (\kappa x)+{C}_{11}\,\cos (\kappa x)+\frac{1}{\kappa }{\int }_{0}^{x}\,\sin (\kappa (x-\xi ))H(\xi )d\xi $$

Finally, the transverse displacement of the beam is obtained by integrating Eq. (49), which is obtained in the form:

60 $$w(x)=\frac{{C}_{3}x-{\beta }_{1}p(x)+{\beta }_{0}{\int }_{0}^{x}\varphi (\xi )d\xi +{\beta }_{1}{\int }_{0}^{x}\theta (\xi )d\xi -{\int }_{0}^{x}(x-\xi ){q}_{z}(\xi )d\xi }{{\beta }_{0}}+{C}_{12}$$

The constants *C*_*i*_ (i.e., *i* = 1, 2, …, 12) can be determined by applying the boundary conditions. Mathematical implementations of the various boundary conditions are expressed as:

Simply Supported Micromorphic Beams:

61 $$\begin{array}{rcl}{u}_{0}(0) & = & {u}_{0}(L)=0\\ w(0) & = & w(L)=0\\ {M}_{xx}(0) & = & {M}_{xx}(L)=0\\ p(0) & = & p(L)=0\\ {\eta }_{xxz}(0) & = & {\eta }_{xxz}(L)=0\\ {\eta }_{xxz}(0)+{R}_{xx}(0)+{R}_{xx,x}(0) & = & {\eta }_{xxz}(L)+{R}_{xx}(L)+{R}_{xx,x}(L)=0\end{array}$$

Clamped-Clamped Micromorphic Beams:

62 $$\begin{array}{rcl}{u}_{0}(0) & = & {u}_{0}(L)=0\\ w(0) & = & w(L)=0\\ \varphi (0) & = & \varphi (L)=0\\ p(0) & = & p(L)=0\\ {p}_{,x}(0) & = & {p}_{,x}(L)=0\\ \theta (0) & = & \theta (L)=0\end{array}$$

Cantilever Micromorphic Beams:

63 $$\begin{array}{ccc}{u}_{0}(0) & = & 0\,{\rm{\& }}\,{N}_{xx}(L)=0\\ w(0) & = & 0\,{\rm{\& }}\,{N}_{xz}(L)=0\\ \varphi (0) & = & 0\,{\rm{\& }}\,{M}_{xx}(L)=0\\ p(0) & = & 0\,{\rm{\& }}\,-{Y}_{xz}(L)+{\eta }_{xxz,x}(L)=0\\  &  & {p}_{,x}(0)=0\,{\rm{\& }}\,{\eta }_{xxz}(L)=0\\ \theta (0) & = & 0\,{\rm{\& }}\,{\eta }_{xxz}(L)+{R}_{xx}(L)+{R}_{xx,x}(L)=0\end{array}$$

### Static analysis of clamped-clamped micromorphic beams

Here, for reasons of simplicity, only the clamped boundary condition is considered at both ends of the beam. To simplify the analysis, the following dimensionless quantities are defined:

64 $$\begin{array}{c}\bar{x}=\frac{x}{L};{\bar{u}}_{0}=\frac{{u}_{0}}{L};\bar{w}=\frac{w}{L};\bar{p}=\frac{p}{L}\\ {\bar{q}}_{x}=\frac{{q}_{x}L}{\mu A};{\bar{q}}_{z}=\frac{{q}_{z}L}{\mu A}\end{array}$$

Here, we assume that the dimensionless axial and transverse distributed loads have the following forms:

65 $${\bar{q}}_{x}={A}_{n}{\bar{x}}^{n}\,{\bar{q}}_{z}={B}_{n}{\bar{x}}^{n}$$

where *A*_*n*_ and *B*_*n*_ are arbitrary constants and *n* is an integer. Using Eq. (65) and substitution of Eq. (64) into Eqs. (47), (48), (54), (55), (59) and (60), we have

66 $${\bar{u}}_{0}(\bar{x})={\bar{C}}_{1}+{\bar{C}}_{2}\bar{x}-\left(\frac{{\alpha }_{1}^{\ast }+{\alpha }_{2}^{\ast }}{{\alpha }_{1}^{\ast 2}+{\alpha }_{0}^{\ast }({\alpha }_{1}^{\ast }+{\alpha }_{2}^{\ast })}\right)\frac{{A}_{n}{\bar{x}}^{n+2}}{(n+1)(n+2)}$$

67 $${s}_{0}(\bar{x})=\left(\frac{{\alpha }_{1}^{\ast }}{{\alpha }_{1}^{\ast }+{\alpha }_{2}^{\ast }}\right){\bar{C}}_{2}-\left(\frac{{\alpha }_{1}^{\ast }}{{\alpha }_{1}^{\ast 2}+{\alpha }_{0}^{\ast }({\alpha }_{1}^{\ast }+{\alpha }_{2}^{\ast })}\right)\frac{{A}_{n}{\bar{x}}^{n+1}}{(n+1)}$$

68 $$\begin{array}{c}\varphi (\bar{x})=\frac{{\alpha }_{1}^{\ast }({\alpha }_{1}^{\ast }+{\alpha }_{2}^{\ast })}{{\alpha }_{1}^{\ast 2}+{\alpha }_{0}^{\ast }({\alpha }_{1}^{\ast }+{\alpha }_{2}^{\ast })}\left(\frac{{B}_{n}{\bar{x}}^{n+3}}{{R}^{2}{\alpha }_{1}^{\ast }(n+1)(n+2)(n+3)}-\left(\frac{{\bar{C}}_{3}}{{R}^{2}{\alpha }_{1}^{\ast }}+\frac{{\bar{C}}_{4}}{{R}^{2}({\alpha }_{1}^{\ast }+{\alpha }_{2}^{\ast })}\right)\frac{{\bar{x}}^{2}}{2}\right)\\ \,+\,{\bar{C}}_{5}\bar{x}+{\bar{C}}_{6}\end{array}$$

69 $$\theta (\bar{x})=\frac{1}{{R}^{2}({\alpha }_{1}^{\ast }+{\alpha }_{2}^{\ast })}\left({R}^{2}{\alpha }_{1}^{\ast }\varphi +\frac{{\bar{C}}_{4}{\bar{x}}^{2}}{2}\right)+{\bar{C}}_{7}\bar{x}+{\bar{C}}_{8}$$

70 $$\bar{p}(\bar{x})=\{\begin{array}{ll}{\bar{C}}_{10}\,\sin (\sqrt{\bar{\kappa }}\bar{x})+{\bar{C}}_{11}\,\cos (\sqrt{\bar{\kappa }}\bar{x})+\frac{1}{\sqrt{\bar{\kappa }}}{\int }_{0}^{\bar{x}}\,\sin (\sqrt{\bar{\kappa }}(\bar{x}-\xi ))H(\xi )d\xi  & {\rm{for}}\,\kappa  > 0\\ {\bar{C}}_{10}\,\sinh (\sqrt{-\bar{\kappa }}\bar{x})+{\bar{C}}_{11}\,\cosh (\sqrt{-\bar{\kappa }}\bar{x})+\frac{1}{\sqrt{-\bar{\kappa }}}{\int }_{0}^{\bar{x}}\,\sinh (\sqrt{-\bar{\kappa }}(\bar{x}-\xi ))H(\xi )d\xi  & {\rm{for}}\,\kappa  < 0\end{array}$$

71 $$\bar{w}(\bar{x})={\bar{C}}_{3}\bar{x}-{\beta }_{1}^{\ast }p(\bar{x})+{\int }_{0}^{\bar{x}}\varphi (\xi )d\xi +{\beta }_{1}^{\ast }{\int }_{0}^{\bar{x}}\theta (\xi )d\xi -\frac{{B}_{n}{\bar{x}}^{n+2}}{(n+1)(n+2)}+{\bar{C}}_{12}$$

where

72 $$\bar{\kappa }=\frac{{\beta }_{1}^{\ast 2}+{\beta }_{1}^{\ast }+{\beta }_{2}^{\ast }}{{\gamma }^{\ast }};R=\frac{1}{L}\sqrt{\frac{I}{A}}$$

73 $$H(\bar{x})=\left({\theta }_{,\bar{x}}+\bar{\kappa }{\int }_{0}^{\bar{x}}\theta (\xi )d\xi -\frac{({\bar{C}}_{4}-{\bar{C}}_{3}{\beta }_{1}^{\ast })\bar{x}}{{\gamma }^{\ast }}-\frac{{\beta }_{1}^{\ast }}{{\gamma }^{\ast }}\frac{{B}_{n}{\bar{x}}^{n+2}}{(n+1)(n+2)}\right)+{\bar{C}}_{9}$$

74 $$\begin{array}{rcl}{\alpha }_{0}^{\ast } & = & \frac{{\alpha }_{0}}{\mu A}=\frac{\lambda }{\mu }+2\\ {\alpha }_{1}^{\ast } & = & \frac{{\alpha }_{1}}{\mu A}=\frac{{\lambda }_{c}}{\mu }+2\frac{{\mu }_{c}}{\mu }-\frac{\lambda }{\mu }-2\\ {\alpha }_{2}^{\ast } & = & \frac{{\alpha }_{2}}{\mu A}=\left(\frac{{\lambda }_{c}}{\mu }+2\frac{{\mu }_{c}}{\mu }-\frac{{\lambda }_{m}}{\mu }-2\frac{{\mu }_{m}}{\mu }\right)\\ {\beta }_{1}^{\ast } & = & \frac{{\beta }_{1}}{\mu A}=\frac{{\mu }_{c}}{\mu }-1\\ {\beta }_{2}^{\ast } & = & \frac{{\beta }_{2}}{\mu A}=\frac{{\mu }_{c}}{\mu }-\frac{{\mu }_{m}}{\mu }\\ {\gamma }^{\ast } & = & \frac{\gamma }{\mu A}=\frac{1}{2}\frac{{\lambda }_{m}}{\mu }\frac{{\ell }_{1}^{2}}{{L}^{2}}+\frac{1}{2}\frac{{\mu }_{m}}{\mu }\frac{{\ell }_{2}^{2}}{{L}^{2}}\end{array}$$

In addition, ${\bar{C}}_{i}$ (*i* = 1, 2, …, 12) are dimensionless constants. Applying the boundary conditions (62), we obtain

75 $${\bar{C}}_{1}={\bar{C}}_{6}={\bar{C}}_{8}={\bar{C}}_{10}={\bar{C}}_{11}={\bar{C}}_{12}=0$$

76 $${\bar{C}}_{2}=\left(\frac{{\alpha }_{1}^{\ast }+{\alpha }_{2}^{\ast }}{{\alpha }_{1}^{\ast 2}+{\alpha }_{0}^{\ast }({\alpha }_{1}^{\ast }+{\alpha }_{2}^{\ast })}\right)\frac{{A}_{n}}{(n+1)(n+2)}$$

77 $${\bar{C}}_{7}=-\frac{1}{{R}^{2}({\alpha }_{1}^{\ast }+{\alpha }_{2}^{\ast })}\left(\frac{{\bar{C}}_{4}}{2}\right)$$

$$\frac{{\alpha }_{1}^{\ast }({\alpha }_{1}^{\ast }+{\alpha }_{2}^{\ast })}{{\alpha }_{1}^{\ast 2}+{\alpha }_{0}^{\ast }({\alpha }_{1}^{\ast }+{\alpha }_{2}^{\ast })}\left(\frac{{B}_{n}}{{R}^{2}{\alpha }_{1}^{\ast }(n+1)(n+2)(n+3)}-\frac{1}{2}\left(\frac{{\bar{C}}_{3}}{{R}^{2}{\alpha }_{1}^{\ast }}+\frac{{\bar{C}}_{4}}{{R}^{2}({\alpha }_{1}^{\ast }+{\alpha }_{2}^{\ast })}\right)\right)+{\bar{C}}_{5}=0$$

$${\bar{C}}_{3}+{\int }_{0}^{1}\varphi (\xi )d\xi +{\beta }_{1}^{\ast }{\int }_{0}^{1}\theta (\xi )d\xi -\frac{{B}_{n}}{(n+1)(n+2)}=0$$

78 $$\{\begin{array}{ll}{\int }_{0}^{1}\,\sin (\sqrt{\bar{\kappa }}(\bar{x}-\xi ))H(\xi )d\xi =0 & {\rm{for}}\,\kappa  > 0\\ {\int }_{0}^{1}\,\sinh (\sqrt{-\bar{\kappa }}(\bar{x}-\xi ))H(\xi )d\xi =0 & {\rm{for}}\,\kappa  < 0\end{array}$$

$$\{\begin{array}{ll}{\int }_{0}^{1}\,\cos (\sqrt{\bar{\kappa }}(\bar{x}-\xi ))H(\xi )d\xi =0 & {\rm{for}}\,\kappa  > 0\\ {\int }_{0}^{1}\,\cosh (\sqrt{-\bar{\kappa }}(\bar{x}-\xi ))H(\xi )d\xi =0 & {\rm{for}}\,\kappa  < 0\end{array}$$

Using the finite difference Newton method, Eq. (78) can be solved to determine the unknown constants ${\bar{C}}_{3}$, ${\bar{C}}_{4}$, ${\bar{C}}_{5}$ and ${\bar{C}}_{9}$. After finding the constants, the problem is completely solved.

It should be mentioned that the micromorphic beam theory reduces to the Timoshenko beam theory by setting ${\mu }_{c}/\mu \to 1$, ${\lambda }_{c}/\lambda \to 1$, as follows:

79 $$\begin{array}{rcl}{\bar{u}}_{0}(\bar{x}) & = & \frac{{A}_{n}}{\left(\frac{\lambda }{\mu }+2\right)(n+1)(n+2)}(\bar{x}-{\bar{x}}^{n+2})\\ \varphi (\bar{x}) & = & \frac{{B}_{n}}{{R}^{2}\left({\left(\frac{\lambda }{\mu }+2\right)}^{2}-\left(\frac{\lambda }{\mu }+2\right)\left(\frac{{\lambda }_{m}}{\mu }+2\frac{{\mu }_{m}}{\mu }\right)\right)(n+1)(n+2)(n+3)}{\bar{x}}^{n+3}\\  &  & -\frac{{\bar{C}}_{1}}{2{R}^{2}\left({\left(\frac{\lambda }{\mu }+2\right)}^{2}-\left(\frac{\lambda }{\mu }+2\right)\left(\frac{{\lambda }_{m}}{\mu }+2\frac{{\mu }_{m}}{\mu }\right)\right)}{\bar{x}}^{2}+{\bar{C}}_{2}\bar{x}\\ \bar{w}(\bar{x}) & = & {\bar{C}}_{1}\bar{x}+{\int }_{0}^{\bar{x}}\varphi (\xi )d\xi -\frac{{B}_{n}{\bar{x}}^{n+2}}{(n+1)(n+2)}\end{array}$$

80 $${s}_{0}(\bar{x})=0;\theta (\bar{x})=0;\bar{p}(\bar{x})=0$$

where $${\bar{C}}_{1}=\frac{{B}_{n}}{(n+1)(n+2)}-{\int }_{0}^{1}\varphi (\xi )d\xi $$; $${\bar{C}}_{2}=-\frac{\mu }{(\lambda +2\mu )}\left(\frac{{B}_{n}}{{R}^{2}(n+1)(n+2)(n+3)}-\frac{{\bar{C}}_{1}}{2{R}^{2}}\right)$$.

## Results and Discussion

**Figure 2:**
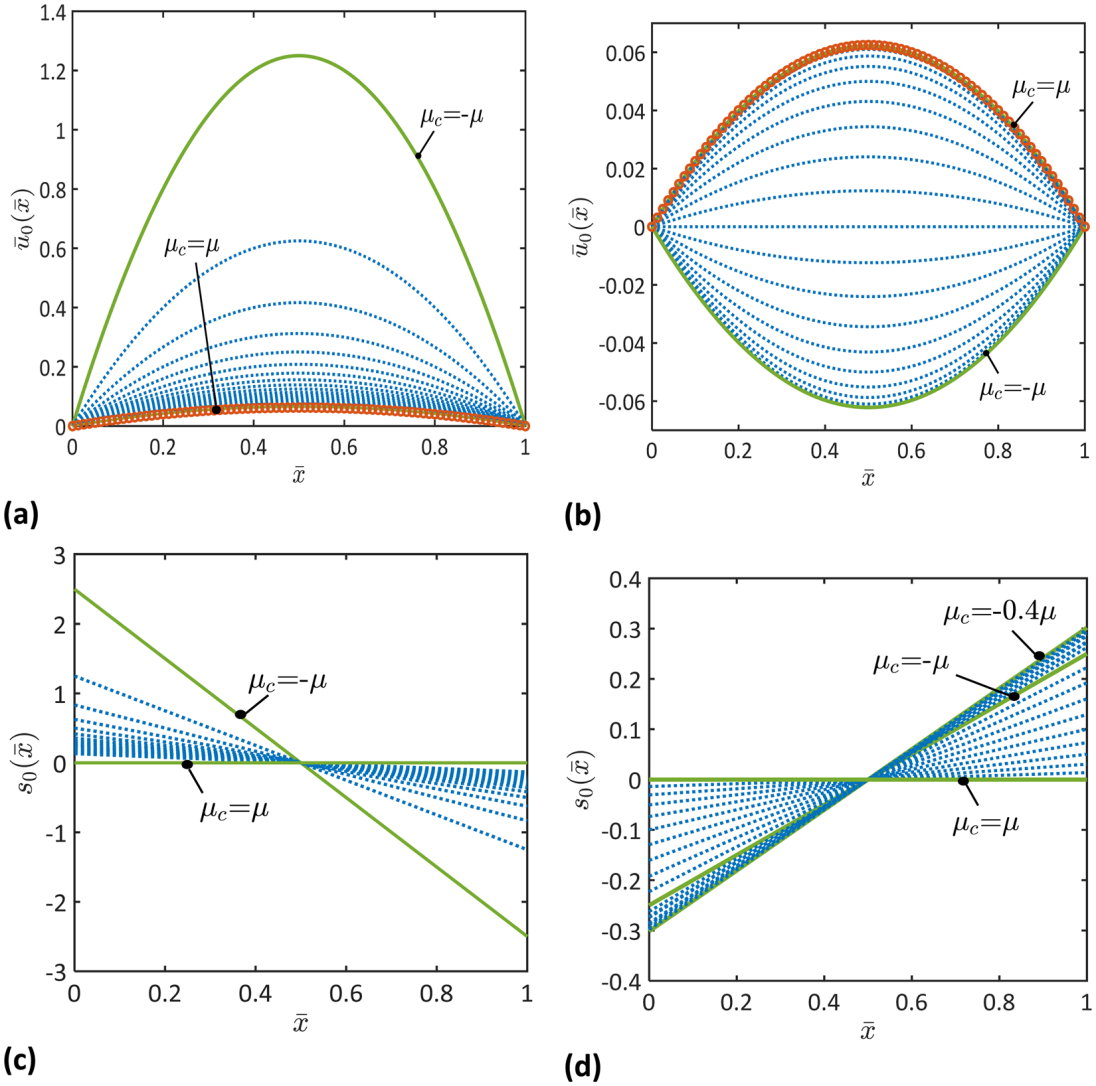
Functions of the nondimensional macroscopic displacement, ${\bar{u}}_{0}(\bar{x})$, and microstrain, ${s}_{0}(\bar{x})$, for different values of the coupling stiffness, *μ*_*c*_, and the microscopic stiffness (**a,c**) ${\mu }_{m}=\mu$ and (**b,d**) ${\mu }_{m}=-\mu$ (${\ell }_{2}=0.8L$, $L/h=100$). Results of Timoshenko beam theory are represented by open circles. Note that the dotted curves represent the evolution of the depicted parameter, as it changes between the solid curves.

**Figure 3:**
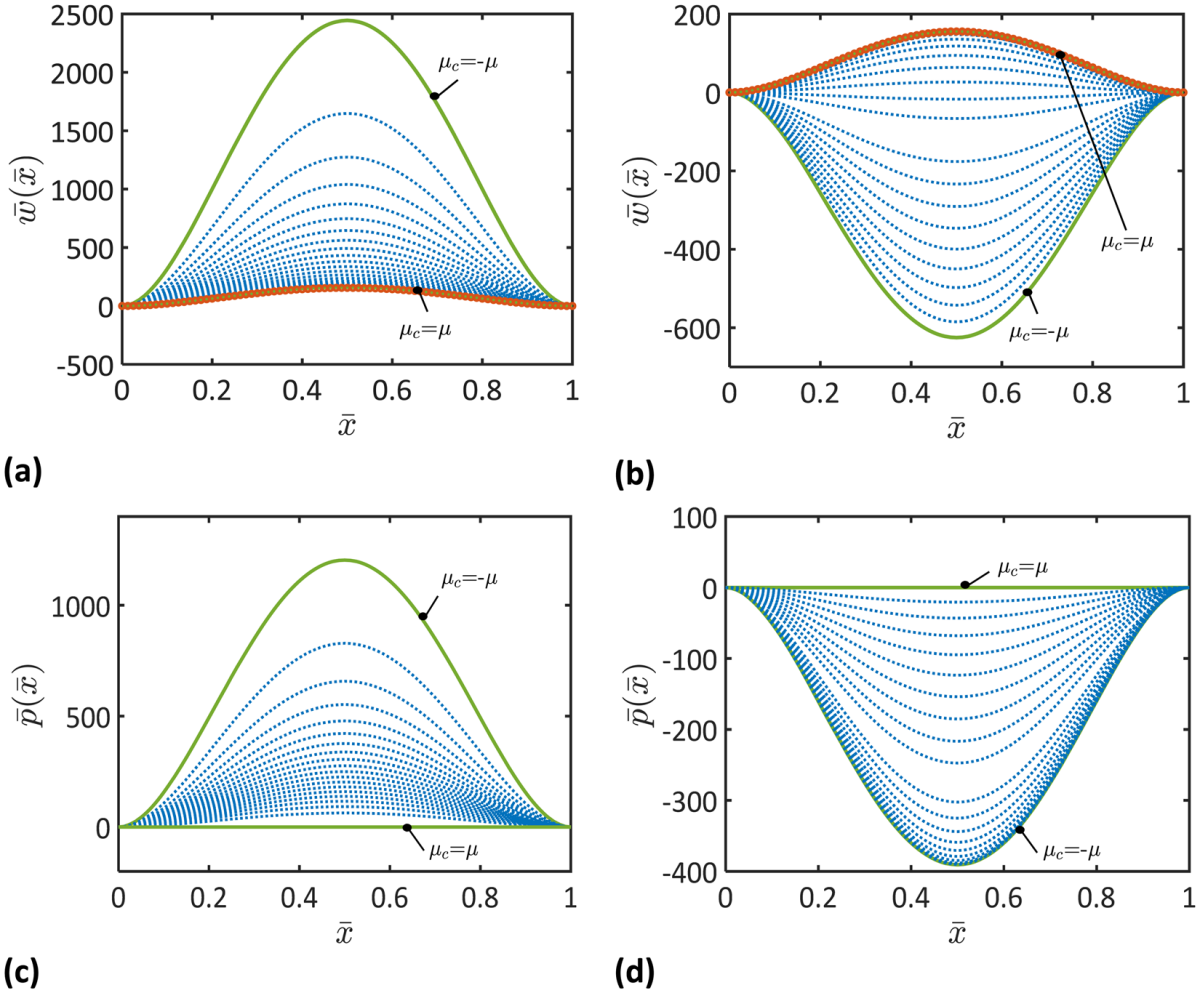
Functions of the nondimensional macroscopic deflection, ${\bar{w}}_{0}(\bar{x})$, and microstrain deflection, ${\bar{p}}_{0}(\bar{x})$, for different values of the coupling stiffness, *μ*_*c*_, and the microscopic stiffness (**a,c**) ${\mu }_{m}=\mu$ and (**b,d**) ${\mu }_{m}=-\mu$ (${\ell }_{2}=0.8L$, $L/h=100$). Results of Timoshenko beam theory are represented by open circles. Note that the dotted curves represent the evolution of the depicted parameter, as it changes between the solid curves.

**Figure 4:**
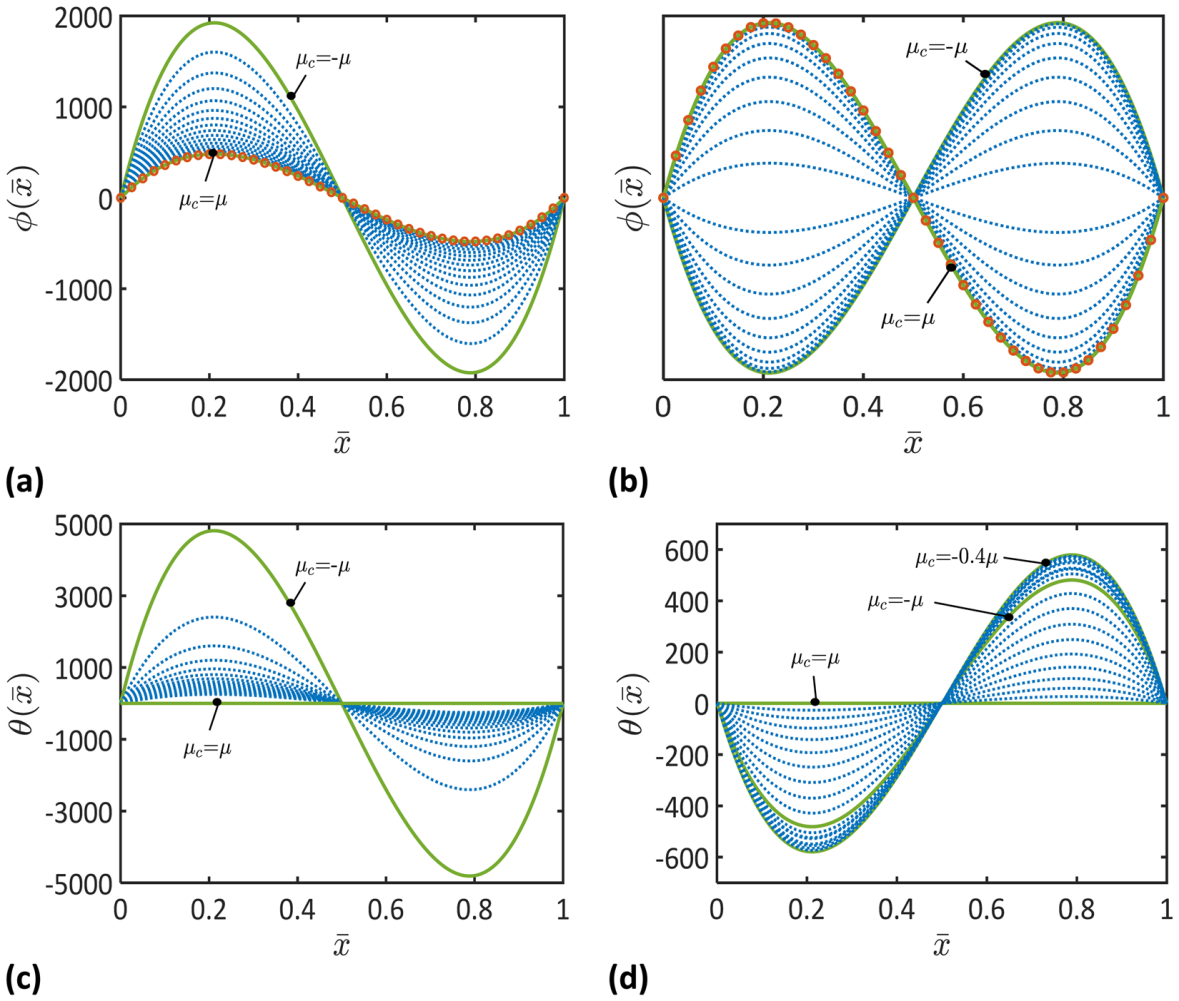
Functions of the nondimensional macroscopic rotation, $\phi (\bar{x})$, and microstrain rotation, $\theta (\bar{x})$, for different values of the coupling stiffness, *μ*_*c*_, and the microscopic stiffness (**a,c**) ${\mu }_{m}=\mu$ and (**b,d**) ${\mu }_{m}=-\mu$ (${\ell }_{2}=0.8L$, $L/h=100$). Results of Timoshenko beam theory are represented by open circles. Note that the dotted curves represent the evolution of the depicted parameter, as it changes between the solid curves.

**Figure 5:**
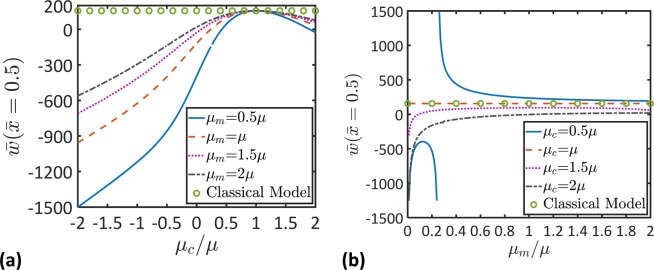
Microstructural topology effects on the nondimensional beam deflection, $\bar{w}$. (**a**) Variation of the nondimensional maximum deflection, $\bar{w}(\bar{x}=0.5)$, as a function of the coupling stiffness, *μ*_*c*_, for different values of the microscopic stiffness, *μ*_*m*_. (**b**) Variation of the nondimensional maximum deflection, $\bar{w}(\bar{x}=0.5)$, as a function of the microscopic stiffness, *μ*_*m*_, for different values of the coupling stiffness, *μ*_*c*_ (${\ell }_{2}=0.8L$, $L/h=100$).

**Figure 6:**
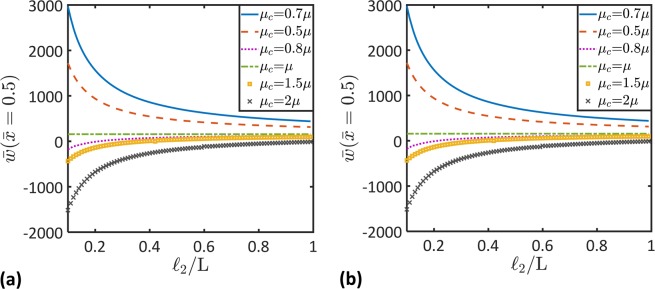
Microstructural topology effects on the nondimensional beam deflection, $\bar{w}$. Variation of the nondimensional maximum deflection, $\bar{w}(\bar{x}=0.5)$, as a function of the length scale, ${\ell }_{2}$, for different values of the coupling stiffness, *μ*_*c*_, and the microscopic stiffness (**a**) ${\mu }_{m}=0.5\mu$ and (**b**) ${\mu }_{m}=2\mu$ ($L/h=100$).

**Figure 7:**
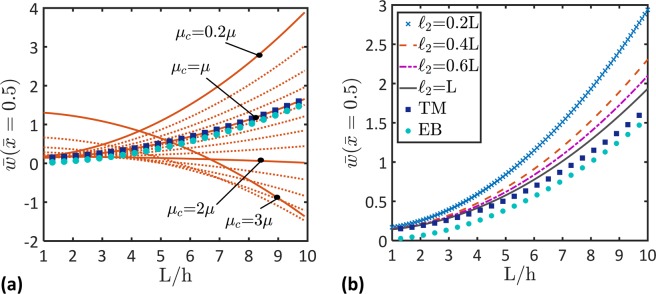
Variation of the nondimensional maximum deflection, $\bar{w}(\bar{x}=0.5)$, as a function of the length-to-thickness ratio, *L*/*h*, for different values of (**a**) the coupling modulus, *μ*_*c*_, (${\mu }_{m}=0.8\,\mu$ & ${\ell }_{2}=0.8L$) and (**b**) the length scale, ${\ell }_{2}$, (${\mu }_{c}={\mu }_{m}=0.8\,\mu$). Results of Timoshenko beam theory (TM) and Euler-Bernoulli beam theory (EB) are also depicted. Note that the dotted curves represent the evolution of the depicted parameter, as it changes between the solid curves.

In this section, the derived analytical solutions of clamped-clamped micromorphic beams are employed to investigate effects of the microstructural topology on the mechanics of beams. To this end, a beam subjected to unit-uniform axial and transverse loads, i.e., $n=0$ and ${A}_{n}={B}_{n}=1$, is considered with the microstructural parameters as given in Table 1. In Figs. 2–4, the variations of the deformation parameters (i.e., ${\bar{u}}_{0}$, $\bar{w}$, $\varphi$, ${s}_{0}$, $\bar{p}$, $\theta$) over the beam length are depicted for different microstructural parameters, *μ*_*m*_ and *μ*_*c*_. In addition, the microstructural topology effects on the nondimensional beam deflection, $\bar{w}$, are depicted in Figs. 5 and 6. Finally, the beam deflection as obtained with the developed micromorphic beam theory is comparison to the ones obtained by Timoshenko and Euler-Bernoulli beam theories in Fig. 7.

**Table 1:** Geometrical and microstructural material parameters as considered in the performed analyses.

Parameter	Value
Microscopic Moduli	${\lambda }_{m}=\lambda =0$
	$${\mu }_{m}=-\mu \to 2\mu $$
Coupling Moduli	${\lambda }_{c}=\lambda =0$
	$${\mu }_{c}=-\mu \to 2\mu $$
Length Scales	${\ell }_{1}=0$ & ${\ell }_{2}=(0.1\to 1)L$
Beam Geometry	$L=(0.1\to 100)h$, where *h* is the beam thickness.

It follows from Fig. 2–4 that the macroscopic and microscopic parameters of the beam deformations, ${\bar{u}}_{0}$, $\bar{w}$, $\varphi$, ${s}_{0}$, $\bar{p}$, $\theta$, would decrease or increase depending on the coupling and microscopic moduli, *μ*_*c*_ and *μ*_*m*_. When ${\mu }_{m} > 0$, each of these parameters decreases as the coupling modulus, *μ*_*c*_, increases from *μ*_*c*_ = −*μ* to *μ*_*c*_ = *μ*. When ${\mu }_{m} < 0$, the macroscopic parameters including ${\bar{u}}_{0}$, $\bar{w}$, and $\varphi$ decrease as the coupling modulus, *μ*_*c*_, increases from ${\mu }_{c}=-\mu$ to *μ*_*c*_ = *μ*. When ${\mu }_{m} < 0$, an increase in the microscopic parameters, ${s}_{0}$, $\bar{p}$, $\theta$, is observed as the coupling modulus, *μ*_*c*_, increases from ${\mu }_{c}=-\mu$ to ${\mu }_{c}=-0.4\,\mu$ followed by a decrease in the microscopic parameters as the coupling parameter increases from ${\mu }_{c}=-0.4\,\mu$ to ${\mu }_{c}=\mu$. These results demonstrate the great influence of the microstructural topology on the deformation of the beam’s microscopic unit cells and the macroscopic deformation of the entire beam.

To demonstrate the role of the microstructure dependence of the beam deformation, the results of the micromorphic beam theory are compared to the results of Timoshenko beam theory in Figs. 2–4. It can be noticed that the developed micromorphic beam theory reduces to Timoshenko beam theory when ${\mu }_{c}=\,{\mu }_{m}=\mu$.

Figures 2–4 indicate that beams with negative equivalent stiffnesses can be made by tailoring their microstructures. For instance, the deflection and axial displacement of a beam with negative microscopic and coupling stiffnesses, ${\mu }_{m} < 0$ and ${\mu }_{c} < 0$, are obtained of negative values in Figs. 2(b) and 3(b). This indicates that the beam deforms in a direction that is opposite to the direction of the applied forces. A beam with such a behavior is of a negative flexural and axial stiffnesses. These results match the recent observations of metamaterials with negative stiffnesses^15–17,46^.

To elucidate microstructural topology effects on the static behavior of micromorphic beams, the variations of the nondimensional maximum deflection, $\bar{w}(\bar{x}=0.5)$, with the coupling modulus, *μ*_*c*_, and the microscopic modulus, *μ*_*m*_, are illustrated in Fig. 5. It is clear that the micromorphic beam model gives the same results as the classical beam model when ${\mu }_{c}=1$. When ${\mu }_{c} < 1$, it is observed that the deflection decreases as *μ*_*m*_ increases. However, when ${\mu }_{c} > 1$, an opposite behavior is obtained. It can also be observed that the beam overall flexural stiffness increases as *μ*_*m*_ increases where the maximum beam deflection decreases as *μ*_*m*_ increases.

Figure 5 shows cases in which the beam would deflect in a direction that is opposite to the direction of the applied load. Beams with such a behavior can be designed by tailoring the microstructure according to the results presented in Fig. 5. It should be mentioned that these designs should be carried out in parallel to analyses of the material stability^17,46–48^ when elastic moduli with negative values would be used.

The effect of the material length scale parameter,$\,{\ell }_{2}$, on the maximum deflection of the beam is displayed in Fig. 6a,b for ${\mu }_{m}=0.5\,\mu$ and ${\mu }_{m}=2\,\mu$, respectively. It is seen that the maximum deflection decreases as the length scale parameter, ${\ell }_{2}$, increases. This can be attributed to the increase in the contribution of the microstrain gradient to the beam deformation as ${\ell }_{2}$, increases. It is also can be noticed that the influence of the microscopic and coupling moduli, *μ*_*m*_ and ${\mu }_{c}$, to the beam deflection decreases as the length scale parameter, ${\ell }_{2}$, increases.

To demonstrate the superiority of the developed micromorphic beam theory over the Timoshenko beam theory (TM) and the Euler-Bernoulli beam theory (EB), the variation of the nondimensional maximum beam deflection ($\bar{w}(\bar{x}=0.5)$) as a function of the beam length-to-thickness ratio (*L*/*h*) is depicted in Fig. 7. The classical beam theories give the maximum beam deflection increases with an increase in the *L*/*h* ratio. However, according to the micromorphic beam theory, the beam deflection would increase or decrease as a function of the *L*/*h* ratio depending on the coupling modulus, *μ*_*c*_, (Fig. 7(a)) and the length scale, ${\ell }_{2}$. It is commonly known that the Timoshenko beam theory is preferred over the Euler-Bernoulli beam theory when *L*/*h* is lower than 20 where the two theories give the same results when *L*/*h* is higher than 20. However, it follows from Fig. 7 that the micromorphic beam theory is preferred over the classical beam theories in general. For advanced beams and for cases that require modeling the beam microstructure effects, the micromorphic beam theory is recommended over the classical theories. Thus, for accurate modeling of advanced beams, e.g., beams made of metamaterials, the developed micromorphic beam theory should be used.

## Conclusions

In this paper, we presented the first attempt to develop a micromorphic beam theory. Existing beam theories – including the Euler-Bernoulli and the Timoshenko beam theories –were developed based on the classical mechanics. These classical beam theories give no information about the beam microstructure, or the effect of its stiffness on the behavior of the entire beam. The developed micromorphic beam theory outweighs the classical beam theories in fully describing the beam deformation in relation to the size, shape, and deformation of its microstructure. In this study, we assumed that the beam was produced by rolling, and, therefore, the grains of its microstructure were elongated. Therefore, six independent degrees of freedom were considered to fully describe the displacements and microstrains of the beam. The implementation of the micromorphic beam theory for the elastostatic behavior of beams revealed that the mechanics of beams strongly depends on the stiffness of the beam microstructure. In addition, it was observed that the micromorphic beam theory can capture many of the exceptional properties of advanced beams. For example, we revealed that by tailoring the microstructure, beams with equivalent negative stiffnesses can be designed. In addition, beams that can deflect in a direction in that is opposite to the direction of the applied load. These results strongly agree with the recent development of metamaterial beams. Thus, the developed micromorphic beam theory can be applied to effectively model the mechanics of advanced beams, e.g., meta-, phononic, and photonic beams.

## Data Availability

The data that support the findings of this study are included in the article.
